# TARGETING THE NLRP3 INFLAMMASOME IN MECHANISMS OF SLEEP DEPRIVATION-INDUCED NEUROINFLAMMATION

**DOI:** 10.1093/geroni/igz038.360

**Published:** 2019-11-08

**Authors:** Chad Smith, Kyle Trageser, Giulio M Pasinetti

**Affiliations:** Icahn School of Medicine at Mount Sinai, New York, New York, United States

## Abstract

The purpose of this study was to determine if inhibitors of innate immunity in microglia could attenuate sleep deprivation (SD)-induced psychological impairment, which involve the assembly and activation of the NLRP3 inflammasome. In the study, CD-1 mice were immune-primed with chronic corticosterone treatment (20 mg/kg IP) for two weeks and were subsequently subjected to one bout of 6 hr SD. Mice were sacrificed immediately afterward to measure cytokine concentrations and caspase-1 activity. We found a significant upregulation of caspase-1 activity in the brain of both mice primed with corticosterone then subjected to sleep deprivation as well as mice only subjected to sleep deprivation (p < 0.01). Increased caspase-1 was NLRP3-dependent as treatment with MCC950 (20 mg/kg IP), an inhibitor of NLRP3 inflammasome assembly, completely attenuated SD-induced caspase-1 activity (p < 0.01). Additionally, in SD mice we observed increased microglia reactivity as quantified by IBA+ cells and an increased number of microglia that had reactive amoeboid morphologies, as measured through immunohistochemistry. The administration of the NLRP3 specific inhibitor MCC950 similarly prevented SD-induced changes in microglia morphology. The study established that consequential effects of SD- induced inflammasome activation and microglia activation could be prevented by a selective NLRP3 inhibitor. Given the preliminary beneficial effects of targeting NLRP3 in SD, future investigations will establish the clinical efficacy of microbiome-derived polyphenolic compounds, which we have shown provide protection against neuroinflammation in models of stress induced psychological impairment, to attenuate SD neuroinflammation by targeting the NLRP3 inflammasome.

